# Supplementary material for: Collaborative intelligence in AI: Evaluating the performance of a council of AIs on the USMLE
Source: PLOS Digit Health. 2025 Oct 9;4(10):e0000787. doi: 10.1371/journal.pdig.0000787 (PMC12510544; doi:10.1371/journal.pdig.0000787)
Supplement: S2 File — (ZIP) [file pdig.0000787.s002.zip › S2 Data Raw data files containing unprocessed question inputs and LLM outputs including transcripts of deliberations/STEP3_Question-91-1_Type-MC-NJ.docx]

**Facilitator** **[Round 1]:** A 22-year-old man comes to the office for evaluation 3 days after his girlfriend was diagnosed with trichomoniasis vaginalis. The patient has not had urethral discharge, dysuria, testicular pain, or genital skin lesions. He has been monogamous with his girlfriend and has been dating her for the past 6 months. He previously had one sexual partner at age 20 years. The patient has not consistently used condoms. He has never had any sexually transmitted diseases. Medical history is unremarkable and he takes no medications. Vital signs are normal. Genitourinary examination discloses no lesions or urethral discharge. Testes are symmetrical without tenderness or masses. There are shotty inguinal lymph nodes. In addition to recommending condom use, which of the following is the most appropriate next step? (A) Obtain a urethral swab for potassium hydroxide preparation (B) Order urinalysis and urine culture (C) Prescribe ceftriaxone and doxycycline therapy (D) Prescribe metronidazole therapy (E) Reassure the patient that no treatment is necessary

**AI_E:** RESTATING THE QUESTION: Given that a young, sexually active man's girlfriend was recently diagnosed with trichomoniasis vaginalis, what is the recommended management course for him, despite showing no symptoms of the infection himself? To further define, Trichomoniasis vaginalis is a sexually transmitted infection (STI) caused by a unicellular, flagellate protozoan parasite. PLANNING: The assumptions are that (1) If a patient's sexual partner is diagnosed with trichomoniasis, the patient may also have the infection, (2) A patient can have trichomoniasis without any symptoms, and (3) If the patient is infected, they will need to be treated. Let's generate alternate assumptions: (A) The patient cannot have trichomoniasis as he shows no symptoms, (B) Even if the patient is infected, he may not need treatment if he is asymptomatic. Assumption (B) should be rejected due to the potential for further transmission of the disease, leading to the hypothesis that the asymptomatic patient should be treated if he has the infection. CLINICAL REASONING: Sexually transmitted infections (STIs) like Trichomoniasis are often asymptomatic, especially in men, posing a risk for transmission. Despite the absence of symptoms in the patient, since his sexual partner has been diagnosed, it becomes essential for him to get tested and treated to prevent the risk of re-infection in the partner and spread to other potential sexual partners in the future. Metronidazole is the treatment of choice for trichomoniasis. CONCLUDING: Considering the nature of Trichomoniasis, the patient's recent sexual history, and the precept of treating asymptomatic patients to combat the spread of STIs, the treatment with metronidazole becomes necessary, ensuring both individuals in the sexual partnership are cleared of the infection. RESPONSE: Thus, the most appropriate next step is to (D) Prescribe metronidazole therapy, irrespective of his asymptomatic status. This is to prevent the further spread of the sexually transmitted infection and secure the health of the community.

**AI_B:** (1) RESTATING THE QUESTION: What is being asked here is essentially what the best next step would be for managing a 22-year-old male who has recently been exposed to trichomoniasis through his girlfriend. His clinical examination shows no specific signs of sexually transmitted infections (STIs) such as urethral discharge, genital skin lesions, or testicular pain. Trichomoniasis is a common sexually transmitted infection caused by the protozoan parasite Trichomonas vaginalis. (2) PLANNING: The crucial assumptions here are that the patient, despite having no symptoms, has been exposed to trichomoniasis and has not been consistently using protection during sexual intercourse. An alternate assumption could be that he has not contracted the infection. However, considering the risk factors like unprotected sex, recent exposure, and the asymptomatic nature of the infection in men, it would be clinically appropriate to assume he might have contracted the infection. (3) CLINICAL REASONING: Trichomoniasis is often asymptomatic in men, which makes clinical diagnosis not feasible. Moreover, it could lead to complications like urethritis, prostatitis, or epididymitis, besides aiding the transmission of other STIs. Therefore, despite the absence of symptoms, it is considered good clinical practice to treat exposed partners due to the high transmissibility of the infection. (4) CONCLUDING: Based on these considerations, the most appropriate next step would be to treat the patient presumptively for trichomoniasis. This would entail prescribing an appropriate antimicrobial therapy, such as metronidazole, which is the recommended treatment for trichomoniasis. (5) RESPONSE: Therefore, the most appropriate next step for this patient would be to treat him for trichomoniasis, despite the absence of symptoms. This aligns best with choice (D) Prescribe metronidazole therapy.

**AI_C:** (1) RESTATING THE QUESTION: A 22-year-old male who has been in a monogamous relationship with his girlfriend for the past 6 months comes for a consultation 3 days after his girlfriend was diagnosed with trichomoniasis vaginalis. He has no symptoms of a sexually transmitted infection and his physical examination is unremarkable. He has had a total of 2 sexual partners and has not been consistent in using condoms. His past medical history is unremarkable, and he is currently not on any medication. The next step in his management, aside recommending condom usage to reduce his risk of contracting sexually transmitted infections, needs to be decided. Trichomoniasis is a sexually transmitted infection caused by the protozoan parasite Trichomonas vaginalis. It's often symptomless in men, making it tricky to diagnose just by clinical symptoms and physical examination alone. (2) PLANNING: Assumptions from the above scenario could be that the patient has not contracted trichomoniasis just because he doesn't present any symptoms, or that the patient might have the infection, but it's asymptomatic. Considering the knowledge that most men with trichomoniasis are asymptomatic, the second assumption seems more clinically relevant. (3) CLINICAL REASONING: Transmission of Trichomonas vaginalis occurs primarily through sexual activity. Given his girlfriend's recent diagnosis and his history of not consistently using condoms, the patient's risk of infection is considerably higher. Despite his lack of symptoms and normal physical examination, he could still be carrying the infection and could potentially spread it to other partners. necessary to treat him if indicated. (4) CONCLUDING: Therefore, although visibly symptomless, it would be clinically appropriate to treat this patient presumptively for trichomoniasis to prevent potential complications and further spread of the infection. (5) RESPONSE: Hence, the most appropriate next step in this case would be to Prescribe metronidazole therapy (D). Metronidazole is an antiprotozoal medication commonly used to treat Trichomonas vaginalis infection, even in asymptomatic individuals who have a known exposure.

**AI_A:** (1) RESTATING THE QUESTION: The question is essentially asking what would be the most appropriate next step in the management of a 22-year-old man who is seeking evaluation because his girlfriend was recently diagnosed with trichomoniasis vaginalis. Even though he has no symptoms or signs of a sexually transmitted disease, he has not been consistently using condoms and has had a monogamous relationship with his currently infected girlfriend for the last 6 months. Trichomoniasis is a sexually transmitted infection caused by a parasite, and it often has no symptoms in men. When symptoms do occur, they typically include burning or itching in the penis, discharge, or discomfort during urination. (2) PLANNING: The assumption implicit in the question is that even asymptomatic sexual partners of infected individuals need to receive treatment, because they are at risk of infection and can transmit the disease to others. By questioning this assumption, I could propose an alternative assumption: that asymptomatic partners do not need treatment but should be counseled on preventive measures, such as condom use, instead. (3) CLINICAL REASONING: Given the persistence of the parasite in the genital tract and the potential for transmission, trichomoniasis therapy is generally recommended for both partners in a couple to prevent re-infection. It's also vital to note that individuals with trichomoniasis are more susceptible to acquiring HIV if they're exposed to the virus, and if a woman with HIV is infected with trichomoniasis, she's more likely to pass the HIV virus on to a sex partner. (4) CONCLUDING: Evaluating the alternative assumptions, it's most likely imminent to ensure that the patient doesn't serve as a reservoir of infection, which can continuously transmit the trichomoniasis back to his girlfriend even after her own treatment. (5) RESPONSE: Thus, the most appropriate next step in managing this patient would be (D) Prescribe metronidazole therapy. Metronidazole is typically the first line of treatment for trichomoniasis, and it's important that his partner also receives treatment to prevent reinfection. It should also be noted that sexual contact should be avoided until both partners have completed their treatment and are symptom-free, to prevent reinfection.

**AI_D:** RESTATING THE QUESTION: The inquiry pertains to a 22-year-old male whose girlfriend has recently been diagnosed with trichomoniasis vaginalis, a sexually transmitted infection caused by the parasite Trichomonas vaginalis. Despite having no symptoms of any sexually transmitted diseases like urethral discharge, dysuria, or testicular pain, he is worried as he has been consistently sexually active with the partner and often without using condoms. His genitourinary examination is largely unremarkable save for shotty inguinal lymph nodes. Given the patient's asymptomatic presentation but potential exposure to Trichomonas vaginalis, the question concerns the most appropriate next step in management. PLANNING: The variables for consideration in planning are the patient's potential exposure to trichomoniasis, his asymptomatic presentation, and the implications of non-treatment. Trichomoniasis is a sexually transmitted infection that may remain asymptomatic in men, potentially causing complications if left untreated. On the contrary, the absence of symptoms may also be due to the patient not being infected. An alternate assumption could be that the patient might have acquired the infection but is currently asymptomatic or in the window period of infection, hence the need to consider treatment to prevent further transmission or complications. CLINICAL REASONING: Trichomoniasis is often asymptomatic in men but they can serve as reservoirs for transmission to their sexual partners. Even in the absence of symptoms, men with exposure should be treated to prevent ongoing transmission. Using this train of thought, the patient's monogamous relationship implies that he might have been exposed to Trichomonas vaginalis from his partner. CONCLUDING: The absence of overt disease-specific features does not preclude the possibility of infection, particularly in the context of reported exposure and a disease known for its often asymptomatic trajectory in males. It is incumbent upon healthcare providers to intercept such potential avenues of continued transmission. RESPONSE: Therefore, I would recommend (D) Prescribe metronidazole therapy. Metronidazole is the first-line treatment for trichomoniasis even if the patient is asymptomatic but has been exposed, thereby potentially halting the further spread of the pathogen.

**Facilitator** The consensus among the AI responses is that the most appropriate next step in managing the 22-year-old man, who has been exposed to trichomoniasis through his girlfriend, is to prescribe metronidazole therapy (D), despite his asymptomatic status. This conclusion is based on the understanding that trichomoniasis, a sexually transmitted infection caused by the protozoan parasite Trichomonas vaginalis, is often asymptomatic in men, making clinical diagnosis challenging. However, untreated, asymptomatic individuals can serve as reservoirs for transmission to their sexual partners. Given the patient's recent exposure and inconsistent use of condoms, it is clinically appropriate to treat him presumptively for trichomoniasis to prevent potential complications and further spread of the infection. Metronidazole is the recommended treatment for trichomoniasis. It is also advised that sexual contact should be avoided until both partners have completed their treatment and are symptom-free, to prevent reinfection.
